# Protocol of a multi-centre randomised controlled trial of a web-based information intervention with nurse-delivered telephone support for haematological cancer patients and their support persons

**DOI:** 10.1186/s12885-015-1314-x

**Published:** 2015-04-17

**Authors:** Jamie Bryant, Rob Sanson-Fisher, William Stevenson, Rochelle Smits, Frans Henskens, Andrew Wei, Flora Tzelepis, Catherine D’Este, Christine Paul, Mariko Carey

**Affiliations:** 1 Public Health/HBRG. HMRI Building, University of Newcastle, Callaghan, NSW 2308 Australia; 2 Department of Haematology, Royal North Shore Hospital, Kolling Institute of Medical Research, University of Sydney, St Leonards, NSW 2065 Australia; 3 The Australian Centre for Blood Diseases, The Alfred Hospital and Monash University, Melbourne, Australia; 4 Priority Research Centre for Health Behaviour, University of Newcastle, Callaghan, NSW 2308 Australia; 5 National Centre for Epidemiology and Population Health, Research School of Population Health, The Australian National University, Building 62 Mills Road, Canberra, ACT 0200 Australia

**Keywords:** Haematological cancer, Psychosocial support systems, Internet, Caregiver, Protocol, Randomised controlled trial

## Abstract

**Background:**

High rates of anxiety, depression and unmet needs are evident amongst haematological cancer patients undergoing treatment and their Support Persons. Psychosocial distress may be minimised by ensuring that patients are sufficiently involved in decision making, provided with tailored information and adequate preparation for potentially threatening procedures. To date, there are no published studies evaluating interventions designed to reduce psychosocial distress and unmet needs specifically in patients with haematological cancers and their Support Persons. This study will examine whether access to a web-based information tool and nurse-delivered telephone support reduces depression, anxiety and unmet information needs for haematological cancer patients and their Support Persons.

**Methods/Design:**

A non-blinded, parallel-group, multi-centre randomised controlled trial will be conducted to compare the effectiveness of a web-based information tool and nurse-delivered telephone support with usual care. Participants will be recruited from the haematology inpatient wards of five hospitals in New South Wales, Australia. Patients diagnosed with acute myeloid leukaemia, acute lymphoblastic leukaemia, Burkitt’s lymphoma, Lymphoblastic lymphoma (B or T cell), or Diffuse Large B-Cell lymphoma and their Support Persons will be eligible to participate. Patients and their Support Persons will be randomised as dyads. Participants allocated to the intervention will receive access to a tailored web-based tool that provides accurate, up-to-date and personalised information about: cancer and its causes; treatment options including treatment procedures information; complementary and alternative medicine; and available support. Patients and Support Persons will complete self-report measures of anxiety, depression and unmet needs at 2, 4, 8 and 12 weeks post-recruitment. Patient and Support Person outcomes will be assessed independently.

**Discussion:**

This study will assess whether providing information and support using web-based and telephone support address the major psychosocial challenges faced by haematological patients and their Support Persons. The approach, if found to be effective, has potential to improve psychosocial outcomes for haematological and other cancer patients, reduce the complexity and burden of meeting patients’ psychosocial needs for health care providers with high potential for translation into clinical practice.

**Trial registration:**

ACTRN12612000720819.

## Background

### Burden of haematological cancers

Haematological malignancies include leukaemias, lymphomas and myelomas. Together, haematological malignancies represent 9% of all cancers in the economically developed regions of the world, and are the fourth most common type of cancer [1]. Five year relative survival rates for haematological malignancies vary depending on a range of factors including the type of cancer and patient age. In Australia, 5-year relative survival rates range from 24% for acute myeloid leukaemia [2] to 87% for Hodgkin lymphoma [2].

### Psychosocial wellbeing of individuals with haematological cancers

Treatments for haematological cancers can include intensive chemotherapy, radiotherapy, surgery and haematopoietic stem cell transplantation [3-6]. Treatments are often urgent and result in debilitating side effects including nausea, depression, fatigue, vomiting, diarrhoea, oral mucositis, ovarian failure and infertility [7-9]. Unlike many other types of cancers, treatment for haematological malignancies often requires long periods of hospitalisation and isolation to prevent infections [10,11]. Given the aggressive and complex treatments required, it is not surprising that levels of cancer-related distress are high. Rates of anxiety amongst haematological cancer patients range from 22% [12] to 27% [13], and rates of depression between 17% [12] and 31% [13]. Unmet needs remain high even after active treatment has ceased, with 27% of haematological cancer survivors reporting unmet needs related to finances, emotional support, and information [14].

### Significant burden placed on support persons

Support Persons are individuals identified by patients as a main source of support in coping with cancer and its treatment. They might be the patient’s spouse, child, other family member, or friend. Support Persons play an essential role in providing emotional and practical support to patients [15], however many report that they have inadequate education and skills to undertake this role [16]. The significant burden placed on Support Persons often results in psychological morbidity, including anxiety and depression, as well as adverse effects on physical health, work and family commitments, and finances [17-19]. Caregivers of haematological cancer patients report needing information about managing side effects and symptoms, and giving medications [16]. Among the most important factors identified by Support Persons of haematological cancer patients for improving their own quality of life are improved communication with health care providers and receiving education and support [16].

### Evidence-based strategies for reducing psychosocial distress

A substantial amount of research has examined strategies to reduce psychosocial distress among individuals with cancer. Although little work has been done with patients diagnosed with haematological cancers, the substantial amount of research with other cancer patient groups suggests psychosocial distress can be minimised by:

#### Ensuring that patients are involved in decision making

Decisional support for treatment has been linked to a number of positive outcomes including greater knowledge, more realistic expectations and a better match between patient values and decisions [20]. There is considerable variation, however, in patient preferences for the extent of involvement in decision making regarding their cancer care [21]. Therefore the ability of clinicians to accurately identify patient preferences for involvement in treatment decisions is critical. Research has shown that in 58% of cases clinicians do not accurately assess patient preferences [21,22]. Effective forms of communication that account for individual factors, such as health literacy and patient preference, are essential to provide patients with an opportunity to take an active role in decision making about their health care.

#### Providing tailored information

The principle of patient-centeredness endorsed by the Institute of Medicine promotes health care that is tailored and responsive to patient needs [23]. Providing patients with information about treatment, including both procedural and sensory information, can reduce patient distress and improve recovery [24-26]. Despite these potential benefits, many cancer patients report high levels of unmet information needs [27]. Guidelines for the psychosocial care of cancer patients recommend that information is tailored to individual circumstances and preferences for amount and type of information [28]. Tailoring is likely to be particularly important for haematological cancer patients and their Support Persons given that haematological cancers are a very diverse group of diseases that often require a range of complex and rapidly changing treatment regimes. Customising diagnosis and treatment information to ensure concordance with the medical circumstances of the individual may help to prevent unnecessary anxiety as a result of viewing information that is not relevant [28]. Providing an opportunity for patients and their families to talk to an experienced cancer nurse can improve understanding and reduce psychosocial morbidity [28]. Patients vary in their preferences for the amount of information they wish to receive, the way the information should be presented and the level of detail they would like to know [29]. Tailoring information to match individual preferences and health literacy can improve psychosocial outcomes and recall of information [23,28,30].

#### Preparing patients for potentially threatening procedures

High levels of anxiety can result in poor recall of information, which has implications for patient adherence to recommendations for care [31]. The prevalence of pre-treatment anxiety is very high among patients undergoing cancer treatment [32]. While there is evidence that preparing patients for potentially threatening medical procedures can reduce anxiety and pain, and improve recovery, information provision prior to treatment is often inadequate [28]. Therefore, there is potential to improve psychosocial outcomes for cancer patients via integration of preparatory information into routine care.

#### Providing psychological therapies

There is substantial evidence for the effectiveness of psychological therapies, including supportive and cognitive-behavioural therapies, for reducing treatment-related distress in cancer patients [28]. Meta-analyses have demonstrated the effectiveness of psychological therapies, finding significant improvements across a range of outcomes for cancer patients including: emotional adjustment, social functioning, quality of life, anxiety, depression, and physical symptoms. [33,34] While there is demonstrated success in improving outcomes for patients who access psychological therapies, the high prevalence of anxiety^12 13^, depression^12 13^ and unmet needs [14] among individuals with cancer suggest there may be barriers to accessing such treatments. Barriers to the use of psychological support services reported by patients include a lack of patient awareness of services, lack of provider referral/endorsement, inconvenient location of services, lack of willingness to talk to a stranger or therapist about personal issues, and difficulty scheduling appointments around treatment and other commitments [35,36].

### The Internet is an ideal platform to provide support which aligns with the evidence base

Most Australians have access to the Internet (83%) [37], and it is frequently reported as an important source for obtaining medical information [38]. There are a number of advantages in using web-based interventions for information provision and psychosocial support: information can be tailored based on user circumstances (e.g. diagnosis), needs and preferences using algorithms; users can exercise control over the type and level of information they receive and the frequency with which they access the information; it allows for real-time customisation based on user needs and preferences (e.g. text size); information can be presented in a range of formats including text, graphics and videos to suit the literacy levels of users, thereby improving understanding and recall; and web-based interventions can incorporate interactive features to facilitate communication and information sharing, such as email and discussion forums. From a service provider perspective, a major advantage of web-delivered interventions is that it enables central delivery of standardised evidence-based information independent of resource constraints of individual health care settings [39]. A web-based approach can also be used to facilitate access to information and support for Support Persons.

### Web-based interventions to improve psychosocial outcomes for patients and their Support Persons

Several reviews have investigated the effectiveness of web-based approaches for patient education and psychosocial support. [40-43] These reviews have demonstrated some evidence in favour of web-based interventions for improving patient outcomes, including knowledge, healthy behaviours, social support and psychosocial wellbeing. However, the conclusions drawn from the literature are limited by mixed findings and methodological weaknesses of the available studies. Furthermore, given that there are no published studies evaluating web-based interventions designed to improve psychosocial outcomes specifically for patients with haematological cancers and their Support Persons, there is a need to further develop this evidence base. The potential for web-based interventions to improve psychosocial outcomes for haematological cancer patients is likely to be enhanced by ensuring that interventions are integrated, tailored to the needs and preferences of the patient, involve Support Persons, provide support for treatment decision making and preparation for treatment, and provide support across the pre-treatment, treatment and post-treatment phases of illness.

### Aims

This study will examine, using a randomised controlled trial, whether an integrated approach that includes access to a web-based information tool and nurse-delivered telephone support reduces for both haematological cancer patients and their Support Persons (i) unmet information needs (primary outcome); and (ii) depression; and (iii) anxiety (secondary outcomes); at 2, 4, 8 and 12 weeks follow up.

### Hypotheses

It is hypothesised that:

(i) Patients who are allocated to the intervention group will have a 0.45 standard deviation lower mean total score on the Health System and Information Needs Domain of the Supportive Care Needs Survey Short Form (SCNS-SF34) (primary outcome), and 0.45 standard deviation lower anxiety and depression scores as measured by the Hospital Anxiety and Depression Scale (HADS) (secondary outcomes), compared to patients who are allocated to the control condition;
(ii) Support Persons who are allocated to the intervention group will have a 0.45 standard deviation lower unmet information needs score as measured by the Support Persons Unmet Needs Survey (SPUNS) (primary outcome), and a 0.45 standard deviation lower depression and anxiety score as measured by the Depression and Stress Scale-21 (DASS) (secondary outcomes), compared to Support Persons who are allocated to the control condition.

## Method

### Study design

This study is a non-blinded, parallel-group, multi-centre randomised controlled trial comparing the effectiveness of a web-based information tool and nurse-delivered telephone support with usual care. The primary outcomes for both patients and Support Persons are unmet information needs and the secondary outcomes are anxiety and depression. All outcomes are assessed a 2, 4, 8 and 12 weeks post-recruitment. This study is funded by a Cancer Institute New South Wales Translation Program Grant (10/THS/2-14).

### Hospital eligibility

Participants will be recruited from the haematology inpatient wards of five Australian hospitals located in New South Wales. These hospitals were selected because they treated at least 15 eligible participants each year.

### Patient and support person eligibility

Patients will be eligible to participate if they are: aged 18 years or older; English speaking; newly diagnosed with acute myeloid leukaemia, acute lymphoblastic leukaemia, Burkitt’s lymphoma, Lymphoblastic lymphoma (B or T cell), or Diffuse Large B-Cell lymphoma; are potentially making a decision regarding treatment; have a life expectancy of 2 months or more as judged by their clinician; and are able to provide informed consent. Those who have commenced cytotoxic chemotherapy will be excluded. The included haematological malignancies were chosen to represent aggressive malignancies that frequently require urgent inpatient treatment. Support Persons of patients are eligible to participate if they are: aged 18 years or older; able to provide informed consent; and are nominated by the patient participant to be an important source of support in relation to the demands of their cancer diagnosis and treatment. Patients and Support Persons are not required to have access to the internet as the intervention will be available to access using iPads provided in hospital.

### Recruitment and consent procedure

A consecutive sample of haematological cancer patients and their Support Persons will be recruited. Recruitment will take place as soon after the time of diagnosis as possible. Patients will be informed about the research by their treating doctor and provided with a study information statement. Participants who are willing to take part will complete a consent form which will be co-signed by their doctor. Consenting patients will be provided with an information package to pass on to their Support Person if they wish to do so. The information package contains a study information statement for the Support Person detailing the research and what participation would involve. Support Persons who are willing to take part in the study will be instructed to complete an enclosed consent form and return it to the research team using the reply paid envelope provided. Patients are eligible regardless of whether they have a participating Support Person. Support Persons are only eligible to participate if they are supporting a cancer patient who has also consented to participate. In the event of the death or withdrawal of a patient participant, the consenting Support Person, if any, will be asked whether they would like to continue participation.

### Randomisation and blinding

Patients will be allocated to either the intervention or control group depending on the week they are recruited, with weeks randomly allocated to intervention or control group across all hospitals throughout the recruitment period. Support Persons will be allocated to the same group as the patient. The random allocation sequence will be generated using an online random number generator distributed by a research team member not involved in participant recruitment. Participants and health care providers will be blind to the allocation sequence. Clinical trials nurses managing the study at each hospital will be informed by the researchers at the start of each week whether participants recruited over the next week are to be assigned to the intervention or control conditions. Due to the nature of the intervention, it is not possible to blind participants or health care providers to condition allocation. Those responsible for conducting analysis of outcomes and interpretation of data will be blind to group allocation.

### Intervention

Participants randomised to the intervention group will receive access to a web-based information tool and nurse-delivered telephone support for the duration of the study.

### Development of web-based information tool

#### Development of content

The web-based information tool was developed by the Health Behaviour Research Group, University of Newcastle, Australia, in collaboration with the Department of Haematology, Royal North Shore Hospital, Australia, and a multi-disciplinary expert advisory group. The content of the intervention web program was developed using patient information resources from the Leukaemia Foundation Australia, the Cancer Council New South Wales and Macmillan Cancer Support (UK). Permission from these organisations was obtained prior to adaptation of materials. All information sources are clearly acknowledged in the web-based information tool. Two expert advisory groups were convened to review and update content and ensure acceptability and consensus among clinical haematological cancer professionals. Expert advisory groups comprised multidisciplinary health care providers, including haematologists, nurses, clinical psychologists, dietitians, infectious disease experts and palliative care coordinators, as well as members of key patient support organizations. The advisory groups were responsible for reviewing all intervention materials for accuracy, completeness, level of detail, and communication style. Feedback was then collated and discussed at the advisory group meetings until consensus was reached regarding the content. Revised versions of the content were then circulated to members for final review and feedback. The final version of the web-based tool includes information on a range of topics, including information about diagnosis, treatment options, what is involved in each treatment, side effects, self-management strategies, impact of cancer on day to day life, available support, complementary and alternative therapies (see Table 1).

**Table 1:** **Web-based information tool content headings**

Domain	Content
1. Introduction	1.1 Introduction
2. My cancer and its causes	2.1a Acute myeloid leukaemia
	2.1b Acute lymphoblastic leukaemia
	2.1c Burkitt’s lymphoma
	2.1d Lymphoblastic lymphoma (B or T cell)
3. What are my treatment options?	3.1 Chemotherapy
	3.1.1 What does chemotherapy involve?
	3.1.1.1 What tests will I require before and during treatment?
	3.1.1.2 How is chemotherapy given?
	3.1.1.2.1 Cannula
	3.1.1.2.2 Central lines
	3.1.1.2.3 PICC lines
	3.1.1.2.4 Lumbar puncture
	3.1.1.3 How long does chemotherapy take?
	3.1.1.4 Where will I have chemotherapy?
	3.1.1.5 Who will be involved in my care?
	3.1.1.6 What are the safety precautions?
	3.1.1.7 How and when will I know if the chemotherapy has worked?
	3.1.2 What are possible side effects?
	3.1.2.1 Effects on the blood and immune system
	3.1.2.1.1 Avoiding infection
	3.1.2.2 Hair loss or scalp problems
	3.1.2.3 Loss of appetite, nausea or vomiting
	3.1.2.4 Constipation or diarrhoea
	3.1.2.5 Fatigue
	3.1.2.6 Itchy skin and other skin problems
	3.1.2.7 Mouth sores
	3.1.2.8 Infertility
	3.1.2.9 When to contact your doctor
	3.1.3 What happens after chemotherapy?
	3.1.3.1 What happens when I go home?
	3.1.3.2 What are the chances of the cancer coming back?
	3.1.3.3 When should I seek medical assistance?
	3.2 Bone marrow transplant
	3.2.1 What is bone marrow?
	3.2.2 What is a bone marrow transplant?
	3.2.3 Is a bone marrow transplant right for me?
	3.2.4 Types of bone marrow transplants
	3.3 Palliative care
	3.3.1 What does palliative care involve?
	3.3.1.1 How long does palliative care take?
	3.3.1.2 Where would I have palliative care?
	3.3.1.3 Who will be involved in my care?
	3.3.1.4 Can my carer access respite care?
	3.3.2 Symptom management
	3.4 No treatment
	3.5 Clinical trials
	3.5.1 What is a clinical trial?
	3.6 Getting a second opinion
4. Complementary and alternative medicine (CAM)	4.1 What is CAM?
	4.1.1 What is scientific evidence?
	4.1.2 Complementary therapies
	4.1.3 Alternative therapies
	4.2 Why do some people with cancer choose CAM?
	4.3 What types of CAM are there?
	4.4 Important safety information
	4.5 What should I ask my doctor about CAM?
	4.6 How do I decide?
	4.7 Costs
	4.8 Finding a complementary therapist
	4.8.1 Professional associations
	4.9 Talking to complementary therapists
5. Impact of cancer and treatment on my life	5.1 Family
	5.2 Work
	5.2.1 Taking time off work
	5.2.2 Financial issues
	5.3 Education/Studies
	5.4 Partner relationships
	5.5 Social life
	5.6 Body image and sexuality
	5.7 Diet
	5.7.1 Coping with eating problems and side effects
	5.7.2 How to gain weight
	5.7.3 Access to a dietitian
	5.7.4 Recipes and snacks
	5.8 Exercise
6. What support is available?	6.1 Emotional support
	6.2 Accommodation
	6.3 Practical support
	6.4 Transport/parking
	6.5 Financial assistance
	6.6 Support for the family
	6.7 Access to wigs
7. Where can I get further information?	7.1 Websites
	7.2 Telephone helplines
	7.3 Clinical cancer nurse
8. Discussion Forum	8. Discussion Forum

#### Development of software

Development of the technical aspects of the web-based tool was undertaken by the Distributed Computing Research Group, University of Newcastle, Australia. Established web design principles [44,45] were employed to ensure that the tool allows the user to extract the desired information as easily as possible. The site is hosted on a secure Apache Tomcat server at the University of Newcastle. A security certificate protects the site and access to the site is via secure HTTP (HTTPS). The site has been written using HTML5, facilitating the provision of multimedia content and reducing the need for special software on participant computers. Some server behaviour was achieved using code written in Java or PHP. Modules adapted from public domain content management (CMS) software have been used to facilitate generation and modification of textual content. Video content uses the capabilities of HTML5. A mySQL database installed on the web server securely stores user-identified audit information that can be later collated.

#### Functionality

**Figure 1:**
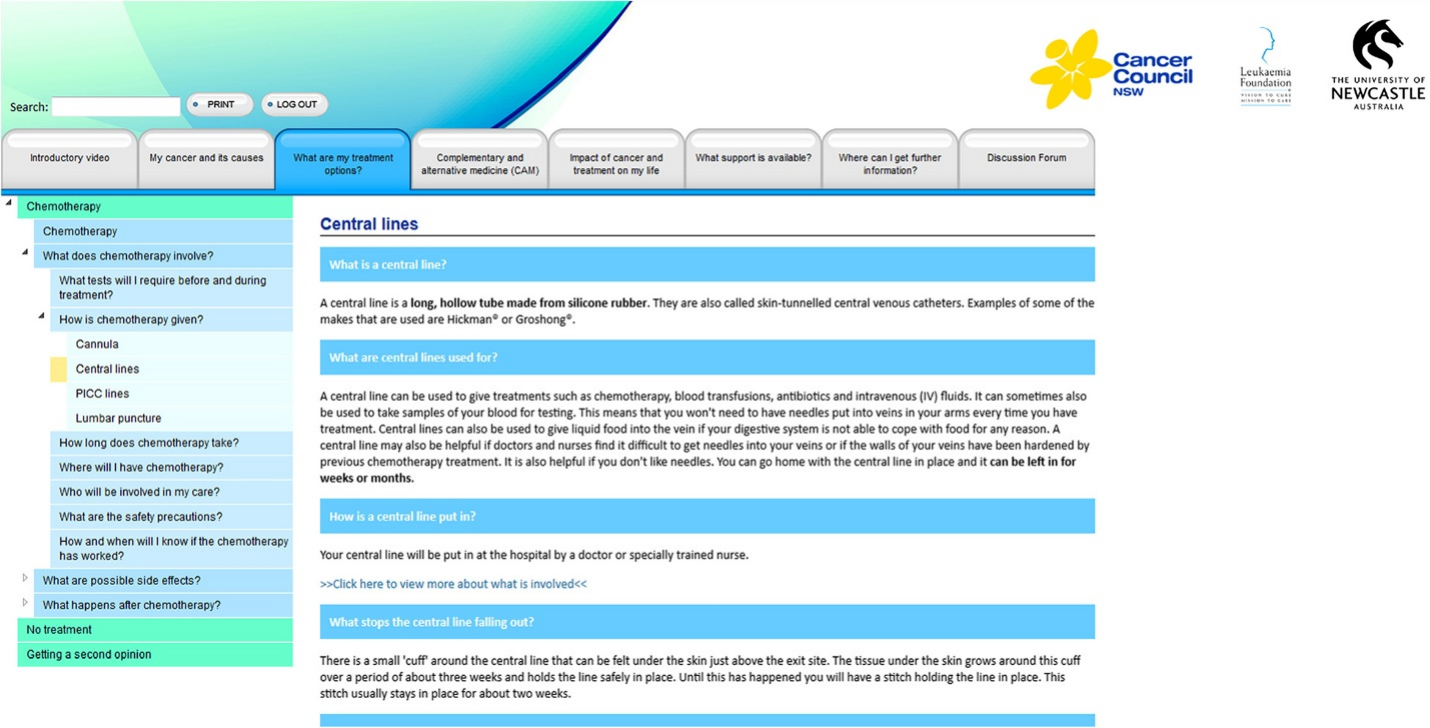
Screenshot of Expandable Text Section.

The information presented in the web-based intervention tool will be tailored to the circumstances of the individual. Participants will only be able to view information that is relevant to their own specific diagnosis and the treatment options that their treating doctor has nominated as suitable for them. Some therapeutic options may be medically contraindicated due to the patient’s health or age at diagnosis. Tailoring to the needs of individuals is further exemplified with information presented in multiple formats, including short videos, text and images to suit different learning styles and literacy levels of participants. Expandable text is provided for sections of information which may be of a sensitive or potentially distressing nature to enable participants to exercise more control over the type of information they wish to view (refer to Figure 1). In addition, explicit categorisation strategies are used to improve recall and understanding, with each section of information summarised into key points.

**Figure 2:**
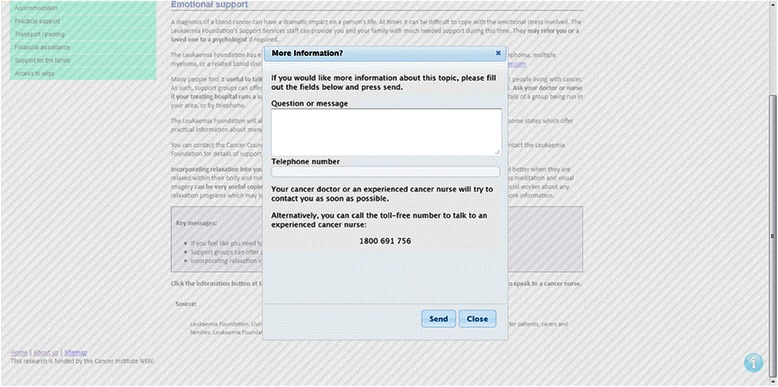
Screenshot of Information Icon.

Participants will be able to search for desired information using the topic tabs, expandable side menus and search function. If participants have additional questions or concerns about any information contained within the web-based intervention, an icon is available at the bottom of every page, which allows the participants to send their questions to the study nurse via automated email (refer to Figure 2). Participants are also able to ask questions and connect with other participants in the intervention group via a discussion forum. Participants are able to create threads on the discussion forum and comment on other users’ threads. The discussion forum is monitored by the lead clinical researcher.

In order to ensure the acceptability and usefulness of the information intervention, a feedback mechanism has been integrated into the web software. Participants in the intervention group will be asked to indicate whether they found the information on the web page they are viewing useful. This data will be collected via a pop-up window and will occur randomly, up to a maximum of 4 times per user. The custom built software will collate data on each user’s access behaviour and feedback.

#### Pilot testing

The penultimate version of the web-based information intervention was pilot tested with a sample of haematological cancer patients (n = 33). Pilot participants were asked to rate sections of information and complete a brief survey about the acceptability of the intervention. Feedback obtained from consumers was evaluated and incorporated if recommended changes reflected the views of the majority. Members of the expert advisory groups were consulted during this revision process to ensure the accuracy of amended information.

### Intervention delivery

#### Web-based information tool

**Figure 3:**
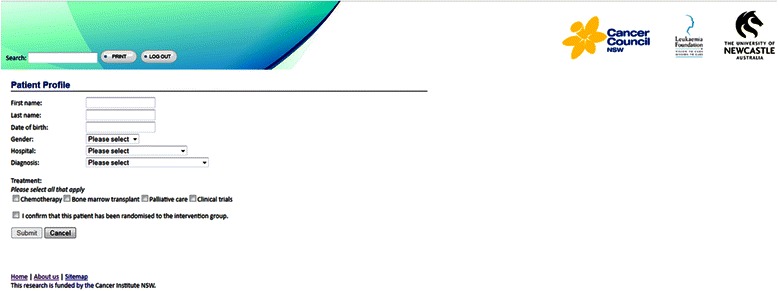
Screenshot of Administrative Interface.

Participants in the intervention group will gain access to the web-based information intervention at the time of recruitment. For consenting patients, the treating doctor will log in to the administrative interface of the intervention website and complete a brief online form indicating the patient’s name, date of birth, gender, diagnosis, and the treatment options that are relevant to the patients’ circumstances (see Figure 3). Using this information the web-based intervention will automatically generate a user ID for the patient. This user ID will be provided to both the patient and their Support Person along with detailed instructions on how to access the web-based information tool both in hospital and at home. The information entered into the online form by the clinician will also be used to tailor the content of the web program.

At least two study iPads (touch screen tablet devices) will be provided to each hospital for use by inpatients who have been assigned to the intervention group to access the web-based information intervention. Outpatients without internet access will also be able to use the iPads to access the intervention while visiting the hospital for outpatient appointments. Support Persons will be able to use the iPads when at hospital, and access the web-based tool using their home internet access. Use of the web-based information intervention will be self-guided, with no restriction placed on how and to what extent it is used. At the time of recruitment, intervention patients will be provided with a demonstration, to supplement the written instructions, on how to use the iPad and access the web-based intervention by a research nurse or study volunteer.

#### Nurse-delivered telephone support

Intervention patients and Support Persons will also have access to an experienced haematological cancer nurse. Participants can contact the cancer nurse via a toll free telephone number to seek information or ask questions. Participants will also have the option of contacting the nurse via the information icon in the web-based information tool, as described above. An automated email will be sent to the nurse providing the patient’s first name, the user’s relationship to the patient, the topic of the page where the icon was clicked, the question/query indicated by the user, the contact telephone number indicated by the user, and details of the treating doctor. The cancer nurse will have experience with addressing information needs and adjustment issues and will be supervised by the lead clinical researcher (WS).

### Usual care

Patients and Support Persons randomised to the control group will receive usual care. The control condition reflects the usual process of psychosocial support and information provision for newly diagnosed haematological cancer patients and their Support Persons. While usual care may vary slightly between participating hospitals and clinicians, psychosocial care and information provision in this early phase of cancer survivorship usually involves the provision of generic written material provided by healthcare providers and non-government patient support organisations such as Cancer Council and/or Leukaemia Foundation. Participants in the control group may seek and obtain information or support that is available on the Internet or from other sources, but will not have access to the web-based intervention or nurse-delivered telephone support specifically developed for this trial.

### Data collection

**Figure 4:**
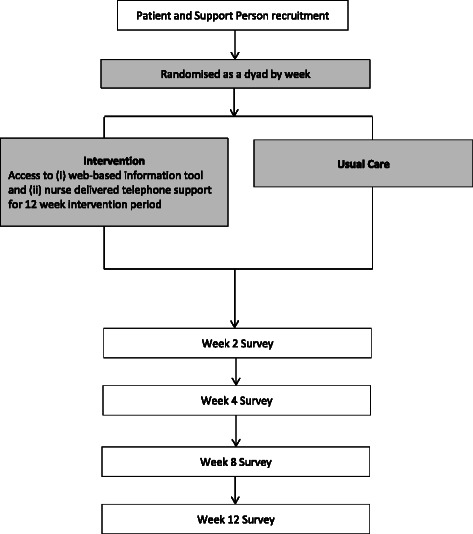
Flowchart of study design.

Outcome data will be collected from patients and Support Persons via paper-and-pen surveys at 2, 4, 8 and 12 weeks post-recruitment (refer to Figure 4). These time points were chosen given the intervention primarily targets the provision of care at diagnosis and during treatment, and it is during this time period when patients are making decisions about and receiving intensive treatment. Data collection for participants who are in hospital will be conducted by research nurses or study volunteers, depending on the preference and policy of the individual hospital. All research nurses and study volunteers will receive comprehensive training. The research nurse or study volunteer will visit the participant in hospital to provide them with the survey package appropriate to the time point and answer any questions about survey completion. Survey packages will be provided to the participant in an unsealed envelope, allowing them to seal their responses prior to returning the survey to the research nurse or volunteer. Participants who do not wish to complete the survey are able to seal the envelope and return it uncompleted. For participants who are not in hospital, including all Support Persons, surveys will be sent via mail with a reply paid envelope to allow return of the completed survey. The study co-ordinator at each participating hospital will be requested to keep an up-to-date record of participants who are discharged or deceased in order to arrange the appropriate method of data collection or remove them from the contact list respectively.

A reminder phone call will be made to non-responders approximately two weeks after the time of the initial delivery of each survey. Reminder phone calls will only occur for participants who are not in hospital. For non-responders who are in hospital, the research nurse or study volunteer will prompt the participant to either complete the survey if they wish to do so or return it uncompleted.

### Outcome measures

Patients and Support Persons in both the intervention and control groups will complete self-report measures of unmet needs, anxiety and depression at 2, 4, 8 and 12 weeks post-recruitment.

#### Patient outcomes

Unmet needs will be assessed using the health systems and information domain of the Supportive Care Needs Survey - Short Form (SCNS-SF34) [46]. The SCNS-SF34 assesses unmet needs across five domains: (i) psychological; (ii) health systems and information; (iii) patient care and support; (iv) physical and daily living; and (v) sexuality. Participants indicate their level of need for each item using a five point scale: ‘no need, not applicable; ‘no need, satisfied’; ‘low need’; ‘moderate need’; and ‘high need’. Domain scores are calculated by summing item responses within the domain. The SCNS-SF34 has high internal consistency for each of the five factors (Cronbach alpha’s 0.86 to 0.96) and good convergent validity [46].

Anxiety and depression will be assessed using the Hospital Anxiety and Depression Scale (HADS) [47]. The HADS is a 14-item self-report instrument which measures anxiety (7 items) and depression (7 items) within the last seven days. Participants respond to each item on a four point scale with 3 indicating that the symptom is severe or frequent, and 0 indicating the symptom is absent or almost absent. Scores from items are summed to provide an overall score out of 21 for the anxiety and depression subscales. The HADS has acceptable internal consistency, construct validity, and discriminant validity [48].

#### Support person outcomes

Unmet needs for Support Persons will be assessed using the information and relationships domain of the Support Persons Unmet Needs Survey (SPUNS) [49], which is a 78-item measure of unmet needs across 6 domains: (i) Information and relationships; (ii) Emotional needs; (iii) Personal needs; (iv) Work and finance; (v) Healthcare access and continuity; and (vi) Worries about the future. Participants provide responses for each item on a five point likert scale from ‘no unmet need’ to 4 ‘very high unmet need’. Domain scores are calculated by summing item responses and dividing by the number of non-missing responses within the domain. The SPUNS has high acceptability, item test-retest reliability, internal consistency (Cronbach alpha = 0.99), and face, content, and construct validity [49].

Anxiety and depression will be measured using the Depression and Stress Scale-21 (DASS-21) [50], a 21-item self-report instrument which measures depression (7 items), anxiety (7 items) and stress (7 items). Participants respond to each item using a four point scale from ‘Did not apply to me at all’ to ‘Applied to me very much or most of the time’. Scores on each scale (Depression, Anxiety, and Stress) are calculated by summing item responses within the scale and multiplying totals by two. Reliability, convergent validity and discriminant validity of the total score and three sub-scales of the DASS-21 is adequate [51,52].

#### Demographic characteristics

Demographic characteristics including date of birth, gender, postcode, marital status, education, time since diagnosis, distance to the cancer centre (for patients only) and relationship to the patient (for Support Persons only) will be collected via participant self-report two weeks post-recruitment.

#### Process measures

Psychological support service utilisation will be examined at 12 weeks post-recruitment for patients and Support Persons in both the intervention and control group. Participants will be asked whether and how many times they have accessed psychological support from a range of services in the past 12 weeks.

Process and acceptability measures (intervention group only): For intervention patients and Support Persons use of the program will be monitored via embedded web monitoring tools. Data collected will include the number of times a participant accesses the web-based intervention, and the type of information accessed will be recorded by the website. Use of the 1800 number will also be monitored via a call log. Acceptability and usefulness of the web-based intervention will be examined via a series of questions in the Week 4 survey for intervention group participants. Clinicians will complete a brief assessment of the perceived acceptability of the intervention at the end of the recruitment period.

### Sample size

Patient sample: Assuming a 75% consent rate, 450 patients will be approached to give a sample of 340 at baseline. Allowing for 15% attrition, at each follow up, a sample of 145 per group will be available at 4 weeks, 123 per group at 8 weeks and 105 per group at 12 weeks. Assuming 90% power and 5% significance level this will enable detection of differences between groups of, 0.38 standard deviations (SD) at 4 weeks, 0.41 SD at 8 weeks and 0.45 SD at 12 weeks follow up. There are over 400 patients diagnosed each year in NSW with the cancers specified in our eligibility criteria. High volume centres will be targeted for recruitment. Current studies being conducted by the group involving clinic-based recruitment are achieving an 85% consent rate. These effect sizes are clinically relevant.

Support person sample: For each of the 340 patient participants, one Support Person will be recruited. It is anticipated that 80% of patients will have a Support Person who provides data at 4 weeks. Allowing for 15% attrition at each follow up, a sample of 115 per group will be available for comparison at 4 weeks, 100 per group at 8 weeks and 85 per group at 12 weeks. Assuming 90% power and 5% significance level this will enable detection of differences between groups of 0.4 SD at 4 week follow up and 0.45 SD at 8 weeks and 0.5 SD follow up. These effect sizes are clinically relevant.

### Statistical analysis

The primary outcome of interest will be scores on the Health Systems and Information Needs Domain of the SCNS-34 (patients), and the information and relationships domain of the SPUNS (Support Persons). Secondary outcomes will be scores on the anxiety and depression subscales of the HADS (patients) and DASS (Support Persons). Outcomes will be compared between groups at each time point using a mixed effects linear regression that includes data from all time points (including baseline), with fixed effects for group, time, the interaction between time and group, and clinic. The model will be adjusted for age and gender (of participant or Support Person as appropriate) and robust variance estimates will be obtained. Treatment group comparisons at each visit will be estimated by differences in least-square means from the group by visit interaction and will be presented as differences in means with 95% Confidence Intervals and accompanying p values. Primary analysis will involve complete case analysis, and sensitivity analysis will be performed using multiple imputation to account for missing data. If the distribution is problematic, we will consider alternative strategies such as a transformation.

### Ethics approval and registration

This study has been approved by the Cancer Institute NSW Clinical Research Ethics Committee in Australia (NHMRC Committee Code: EC00414) (AU RED Reference: HREC/11/CIC/24), the Hunter New England Human Research Ethics Committee (NHMRC Committee Code: EC00403) (AU RED Reference: HREC/13/HNE/338) and The University of Newcastle Human Research Ethics Committee (NHMRC Committee Code: EC00144) (Reference No: H-2012-0011). The study has also been approved by the local research governance committees of each participating hospital. Registration number of the Australian New Zealand Clinical Trials Registry is ACTRN12612000720819, registered on 5/7/2012.

## Discussion

Despite the challenges faced by patients with haematological malignancies and their Support Persons, few interventions have addressed the psychosocial needs of this group. Currently, there is a lack of up to date, comprehensive sources of information for individuals with blood cancer and their Support Persons that covers the entire journey from diagnosis into survivorship. This study will assess whether a web and telephone based approach to providing information and support addresses psychosocial challenges faced by haematological patients and Support Persons. The intervention includes Support Persons, uses technology to support tailoring, is designed with integration into routine clinical practice in mind, and draws on best practice recommendations for the provision of information. The approach, if found to be effective, has potential to improve psychosocial outcomes for haematology patients and other cancer patients, reduce the complexity and burden of meeting patients’ psychosocial needs for health care providers and has high potential for translation into clinical practice.
